# Comparison of in-gel and in-solution proteolysis in the proteome profiling of organ perfusion solutions

**DOI:** 10.1186/s12014-023-09440-x

**Published:** 2023-11-15

**Authors:** Corinna M. Snashall, Chris W. Sutton, Letizia Lo Faro, Carlo Ceresa, Rutger Ploeg, Sadr ul Shaheed

**Affiliations:** 1 Nuffield Department of Surgical Sciences, University of Oxford, Oxford, UK; 2 School of Biosciences, The University of Sheffield, Sheffield, UK; 3 Institute of Cancer Therapeutics, University of Bradford, Bradford, UK; 4 Leiden University Medical Centre, Leiden University, Leiden, Netherlands; 5 Oxford University Hospital NHS Foundation Trust, Oxford, UK; 6 NHSBT Oxford Blood Donor Centre John Radcliffe Hospital, Headley Way, Headington, Oxford, OX3 9BQ UK

**Keywords:** Perfusate, Organ transplantation, Proteomics, Kidney, Liver

## Abstract

**Purpose:**

The organ perfusion solution (perfusate), collected at clinically and temporally significant stages of the organ preservation and transplantation process, provides a valuable insight into the biological status of an organ over time and prior to reperfusion (transplantation) in the recipient. The objective of this study was to assess two bottom-up proteomics workflows for the extraction of tryptic peptides from the perfusate.

**Experimental design:**

Two different kinds of perfusate samples from kidney and liver trials were profiled using liquid chromatography–mass spectrometry (LC-MS/MS). The preparation of clean peptide mixtures for downstream analysis was performed considering different aspects of sample preparation; protein estimation, enrichment, in-gel and urea-based in-solution digestion.

**Results:**

In-solution digestion of perfusate allowed identification of the highest number of peptides and proteins with greater sequence coverage and higher confidence data in kidney and liver perfusate. Key pathways identified by gene ontology analysis included complement, coagulation and antioxidant pathways, and a number of biomarkers previously linked to ischemia-reperfusion injury were also observed in perfusate.

**Conclusions:**

This study showed that in-solution digestion is a more efficient method for LC-MS/MS analysis of kidney and liver organ perfusion solutions. This method is also quicker and easier than in-gel digestion, allowing for greater sample throughput, with fewer opportunities for experimental error or peptide loss.

**Supplementary Information:**

The online version contains supplementary material available at 10.1186/s12014-023-09440-x.

## Introduction

In recent years, focus of research in organ preservation and transplantation has turned from the use of the Static Cold Storage method of organ preservation to a range of non-static machine perfusion techniques. These new methods aim to limit the damaging effects of cold ischaemia in order to keep grafts healthy and viable for longer, and maximise the chances of successful post-transplant outcomes [1]. Machine perfusion requires the use of perfusates: chemically optimised organ preservation solutions containing sugars, nutrients, electrolytes and other supplements designed to maintain cellular metabolism and keep other physiological parameters, such as pH and osmolality, within their natural range so that the organ can remain healthy, functional and protected from ischaemia throughout the preservation period [1]. Perfusion machines provide a controlled flow of perfusate through the organ in a similar way to how the circulatory system would transport blood through the vasculature prior to the organ’s removal [2]. A number of recent and ongoing studies have explored various lengths of time, methods of perfusion, and compared specific perfusate compositions in order to identify the most appropriate perfusion procedures for the types of organ undergoing transplant and the clinical variables associated with donors and recipients [3–7]. The nature of the perfusion process makes perfusate a useful non-invasive and easily accessible source of molecular information as it can be collected at set points of the preservation process to give a temporal reflection of the proteins being secreted by the organ throughout the full length of the perfusion [8].

In recent years and with advances in technology, mass spectrometry (MS) has moved beyond the basics of protein identification and quantitation and into a newer field of clinical applications including diagnostics, biomarker discovery and predictive medicine [9]. Proteomic analysis of clinical samples such as tissues and bodily fluids, known as clinical proteomics, has become a useful and increasingly popular way to obtain information about the health or disease status of a biological system [9]. This is notably the case in the context of organ transplantation, where proteomic profiling of blood, urine, tissue and perfusion fluid has the potential to inform on the physiological status and cellular activity of a donated organ throughout different stages of the transplant process [10]. By observing changes in these profiles, it becomes possible to identify biomarkers that may reflect the impact and effectiveness of different methods of organ preservation or clinical interventions, determine organ quality and even predict transplant outcome [11].

There are, however, additional challenges that can make proteomic analysis of perfusate more difficult in comparison to other types of biological material. The nature of perfusate, being a commercially made solution, means it contains substances not typically found in human samples and is also often supplemented with antibiotics and anti-coagulants prior to perfusion, at the discretion of the clinician [12, 13]. These components can interfere with the standard methods of protein estimation and sample preparation and therefore protocols need to be optimised to take this into account. A further challenge associated with the proteomic analysis of perfusate is the high dynamic range of protein concentrations present within individual samples [14]. This can be a result of clinical variations in donors, but is more significantly affected by the range of perfusion durations, which can vary by many hours between perfusions, and can make comparisons of different samples collected at the end of the perfusion process challenging. Another common obstacle in the field of clinical proteomics is the masking of low abundance proteins in a sample by high abundance proteins such as albumin and immunoglobulins [15]. This is particularly the case in blood-derived samples such as serum or plasma and is also seen in perfusate [10]. For this reason, sample preparation for LC-MS/MS analysis must include steps to reduce the complexity of the material. This often involves employing methods including centrifugation, filtration and solvent precipitation prior to digestion to aid the removal of abundant proteins and/or enrichment of those that are less abundant, though this can still lead to the loss of proteins of interest [15].

Finally, it is important to choose an appropriate method of proteolytic digestion: this is a key step of sample preparation for bottom-up proteomics and involves the digestion, usually using trypsin, of proteins into peptide fragments with specific m/z values which can undergo LC-MS/MS analysis [16]. The two most commonly used approaches are in-gel and in-solution digestion.

In-gel digestion involves using gel electrophoresis to separate the proteins in a sample by molecular weight prior to digestion. After staining, bands are excised by hand and and digested. Benefits include the ability to split complex samples into multiple groups, which can each be analysed individually. This simplifies the sample contents and increases the depth of analysis possible, and the gel separation can also help remove impurities and contaminants [17]. However, the process is lengthy and error-prone, and peptide yield can vary depending on protein properties, gel composition and other factors. The alternative to gel digestion is in-solution digestion. Here, samples are not pre-separated and are instead reduced, alkylated and digested whilst remaining in buffer. This process is quicker, less prone to human error and minimises sample loss, but does risk the inclusion of contaminants unless a desalting step is carried out after digestion. Given the benefits and drawbacks of both methods, it is important to establish the best choice according to sample type.

There has been relatively little reported on the proteomic profiling of perfusate, and as such there is no standardised sample preparation method for samples due to undergo MS analysis.This study aimed to develop a sensitive, specific and reliable method for studying the proteome of perfusate in an unbiased and untargeted way. Optimisation work was performed to identify the most effective methods of sample purification and enrichment, protein estimation and protein digestion, specifically in relation to perfusate as a biological material. It is expected that having a standardised preparation method will broaden the scope of future experiments and provide opportunity for further studies into this area of transplant proteomics.

## Materials and methods

### Study participants and sample collection

**Fig. 1:**
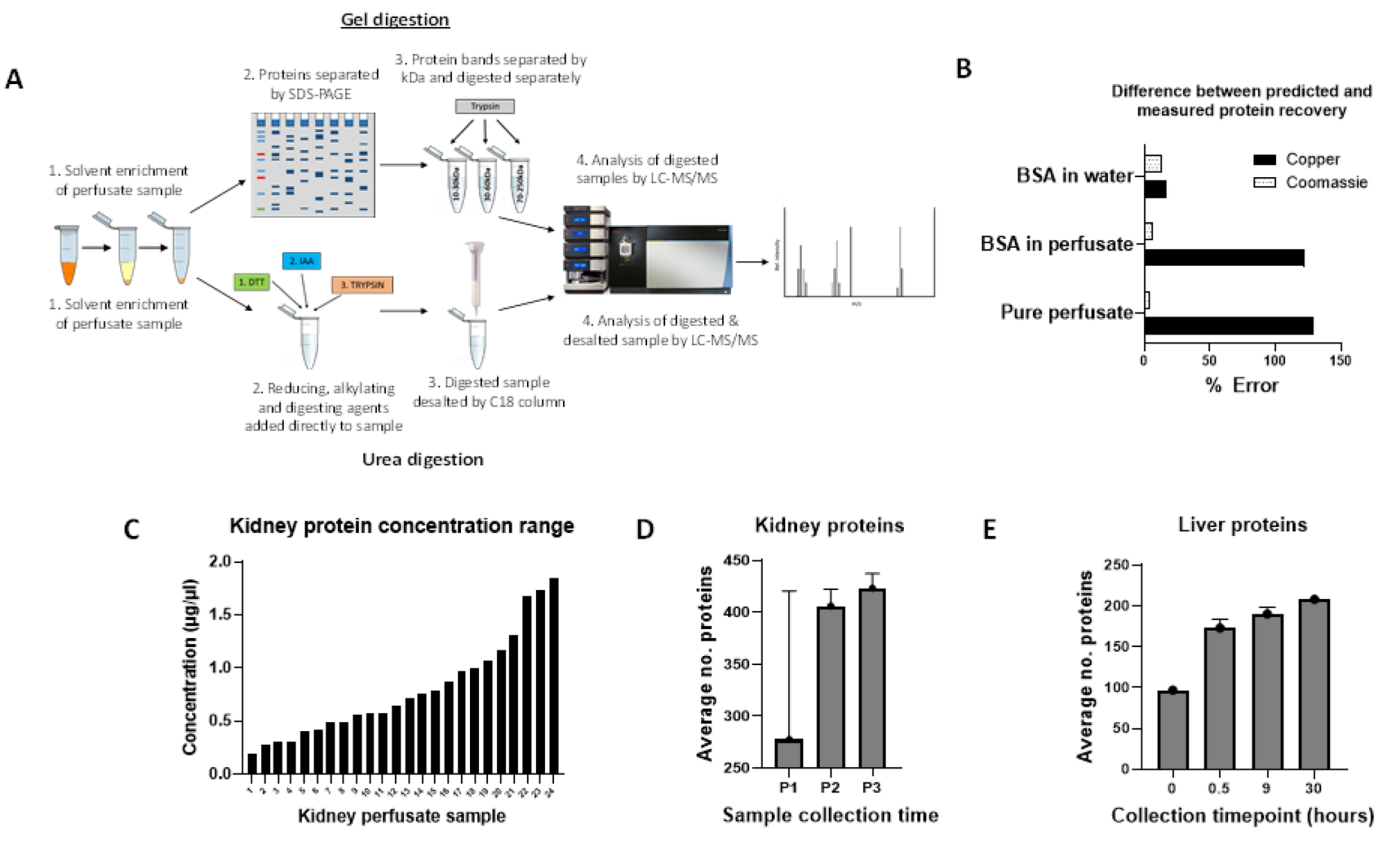
(**A**) Workflow for in-gel and in-solution methods of sample preparation. (**B**) Comparing accuracy of copper- and Coomassie-based assays for correctly measuring known quantities of protein in water and UW-MPS. Predicted and measured concentrations from 5 experiments were normalised using average and standard deviation, and percentage error calculated (see methods). (**C**) protein concentrations of all kidney perfusate samples that underwent Coomassie-based protein estimation assay (n = 24). (**D**&**E**) Average numbers of proteins identified in the kidney (n = 8) and liver (n = 6) perfusate samples that underwent in-solution digestion and LC-MS/MS analysis, by collection timepoint

Perfusate samples used for this study were obtained from the COPE COMPARE kidney study (ISRCTN32967929, NHS Health Research Authority (Ethics Ref number: 14/SC/1056)) and Liver Defatting Study (Ethics Ref: 16/NE/0248). Belzer MPS® Organ Preservation Solution (Bridge to Life Ltd, Northbrook, Illinois, USA) was used for the COPE COMPARE trial while for the liver defatting study, the warm perfusion solution was composed of packed red blood cells and 4% Gelaspan (B Braun, Sheffield, UK), with 10% calcium gluconate (B Braun, Sheffield, UK), 10,000 IU unfractionated heparin sodium (Wockhardt UK Ltd, Wrexham, UK) and 750 mg of cefuroxime (Flynn Pharma Ltd, Dublin, Ireland) added to the reservoir during device priming. Kidney perfusate samples were taken 15 min after the start of perfusion, during perfusion, specifically immediately before the organ left the donor centre (average 7.9 h) and at the end of perfusion, immediately before transplant (average 10.8 h). These timepoints were subsequently referred to as P1, P2 and P3. During liver perfusion the following components were also infused: sodium bicarbonate (B Braun, Sheffield, UK), 25,000 IU unfractionated heparin sodium (Wockhardt UK Ltd, Wrexham, UK), 200 units insulin (Actrapid) (Novo Nordisk, West Sussex, UK), 4.5 g sodium taurocholate (OrganOx Ltd, Oxford, UK), 0.5 mg eporostonol sodium (Flolan) (GLAXO Group Limited, Middlesex, UK), sodium chloride and Nutriflex Special (B Braun, Sheffield, UK). Samples consisting of 1ml perfusate were centrifuged at 13,000rcf for 15 min upon collection and the supernatant was transferred into new tubes and stored long term at -80^o^C until required for analysis. Liver perfusate samples were taken at 0, 0.5 and 8 h for one liver and at 0.5, 10 and 30 h for the other.For the purposes of comparing perfusate composition by timepoint, proteomic data obtained from both 0.5 h samples was combined (T0.5) as was the data from the 8 and 10 h samples (hereafter referred to as T9). Synchronizing sample collection at exactly the same timepoint was difficult, given the nature of the organ donation process which often involves donor and recipient centres in different locations, so we considered that these timepoints were close enough to each other to be comparable and a reasonable “midpoint” of the total perfusion time (30 h). Details of donor characteristics and perfusion durations are provided in Table 1A. An overview of the workflow is provided in Fig. 1A.

**Table 1:**
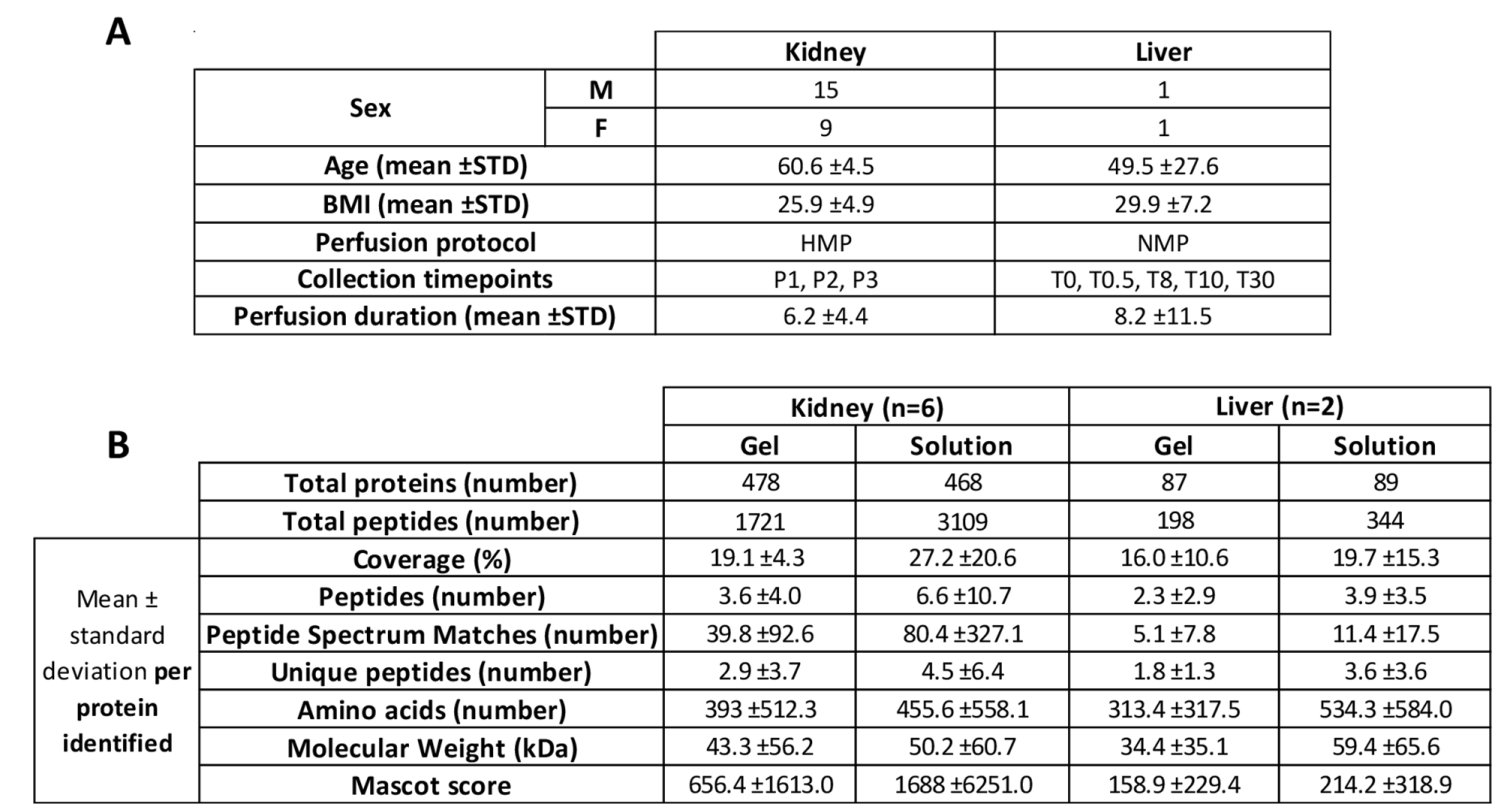
**A**: available donor characteristics for all kidneys (n = 24) and livers (n = 2) used in this study. BMI = weight (kg)/height (cm)^2^. **B**: Comparing characteristics of peptides and proteins identified by LC-MS/MS analysis of the kidney (n = 6) and liver (n = 2) perfusate samples which underwent both in-gel and in-solution digestion

### Protein estimation and enrichment

To select the right protein estimation assay for perfusate, test samples were prepared containing undiluted Belzer MPS® Organ Preservation Solution (UW-MPS), Bovine Serum Albumin (BSA) diluted in UW-MPS and BSA diluted in ultrapure Milli-Q water. The concentration of these samples was measured by copper-based (Pierce™ BCA Protein Assay Kit, Thermo Fisher Scientific, Waltham, MA, USA) and Coomassie-based colorimetric assays (Bradford Reagent, Merck Life Science Limited, Dorset, UK), according to manufacturers’ instructions. Absorbances were measured using iMark Microplate Absorbance Reader (Bio-Rad Laboratories, Hercules, CA, USA). Predicted and measured concentrations in pure UW-MPS and BSA-spiked samples were calculated in triplicate and colorimetric assay data were normalised using the average and standard deviation of the results (normalised = (actual value – average)/standard deviation). Normalised data were used to calculate a percentage error, giving a measure of how close the measured values were to the expected (% error = ((predicted – measured)/measured) x100).

After protein estimation, equal amounts of protein from the perfusate samples (10 µg) were enriched with either HPLC grade ethanol (Merck Life Science UK Limited, Dorset, UK) as described in [18] or HPLC grade acetone (Merck Life Science UK Limited, Dorset, UK) as described in [19] for LC-MS/MS analysis. In short, solvents were added to perfusate samples, mixed and left at -20 °C for 2 h or overnight before centrifuging at 17,000rcf for 20 min at 4 °C. Supernatant was removed, the pellet dried and resuspended in 8 M urea (Merck Life Science UK Limited, Dorset, UK) prior to in-solution digestion. Solvent enrichment was only used for samples undergoing In-solution digestion, as the process of SDS-PAGE separation provides a similar purification effect.

### Gel digestion

Perfusate sample volumes equivalent to 10 µg of protein were mixed with Tris-Glycine SDS Sample Buffer (Thermo Fisher Scientific, Waltham, MA, USA), heated at 60 °C for 10 min, and the proteins separated by size using sodium dodecyl sulfate polyacrylamide gel electrophoresis (SDS-PAGE). Gels were rinsed and stained with Coomassie Blue dye for 30 min, then placed in destain solution consisting of methanol, acetic acid and water and incubated at room temperature with agitation, changing the solution several times, until excess dye was washed out and only the protein bands were visible. Gel digestion was performed similar to the methods described by Shevchenko et al. [17]. In short, following Coomassie staining, each lane was divided into 3 segments with a clean scalpel blade, using the protein ladder for guidance. Lanes were split into sections of proteins with molecular weights of 70-250 kDa, 30-60 kDa and 10-30 kDa. The strongly stained band of albumin (65 kDa) was intentionally excluded and the three pieces were cut further into strips to increase surface area for trypsin digestion. Gel pieces underwent 2–3 alternate washes in 100% acetonitrile (ACN) and 25mM ammonium bicarbonate (AMBIC) to remove residual dye and were incubated in 10ng/µltrypsin (Thermo Fisher Scientific, Waltham, MA, USA) overnight at 37 °C. Digested proteins were extracted from the gel by 2–3 more alternating washes in 100% ACN/25mM AMBIC, with the peptide-containing solution collected and stored on ice before lyophilising and storing at -80 °C until LC-MS/MS/MS analysis. 6 kidney perfusate samples underwent in-gel digestion.

### In-solution digestion

For the in-solution digestion, enriched protein pellets were resuspended in 20 µl 8 M urea in 400mM AMBIC, reduced with 50mM dithiothreitol (DTT) at 70^o^C for 15 min and alkylated using 100mM iodoacetamide (IAA) at room temperature in the dark for 20 min. Urea concentration was then diluted from 8 M to 2 M with 400mM AMBIC in 10% ACN, creating an optimal environment for proteolytic digestion. MS-grade trypsin (Thermo Fisher Scientific, Waltham, MA, USA) was added at a protease-to-protein ratio of 1:30 (w/w) at 37 °C for 3 h. A second step digest was applied after 3 h (giving a final protease-to-protein ratio of 1:60) and incubated overnight at 37 °C. Following digestion, each sample was desalted using BondElut C18 columns (Agilent, Santa Clara, CA, USA), lyophilized and stored at -80^o^C until analysis (Fig. 1A). Kidney perfusate samples (n = 8) were prepared using in-solution digestion, 6 of which were also used for in-gel digestion. Therefore only 6 samples were used to compare digestion methods, while all 8 were used later in the study for proteome interpretation.

### LC-MS/MS analysis

LC-MS/MS analysis was performed on a Dionex Ultimate 3000 HPLC system (Thermo Fisher Scientific, Waltham, MA, USA) coupled to a Orbitrap Fusion mass spectrometer (Thermo Fisher Scientific, Waltham, MA, USA). Lyophilised peptides were reconstituted in solvent A (2% ACN/0.1% FA) and then loaded onto a trap column packed with 5 μm silica particles, 100Å pore size. For in-solution analysis, 4 µg of digested material was loaded onto the analytical column. In the case of in-gel digestion, 10 µg of total protein was loaded on the gel, and this material was subsequently analysed as three separate injections: Sample 1 (70-250 kDa), Sample 2 (30-60 kDa), and Sample 3 (10-30 kDa). After a 4 min wash with solvent A at 15 µl/minute, samples were loaded on an Acclaim PepMap100™ NanoViper column (25 cm× 75 μm ID, 2 μm particle, Thermo Fisher Scientific, Waltham, MA, USA). The peptides were eluted with an 80-minute linear gradient: 2–90% solvent B (90% ACN, 0.1% FA), followed by 8 min column wash with 90% solvent B and 16 min column equlibration with solvent A at 300nL/minute. The Orbitrap Fusion mass spectrometer was operated in data-dependent mode (DDA). The spray voltage was 2.4 kV with an Ion transfer tube temperature of 275 °C and no sheath gas was used for the setting of the nano electrospray ion source. The MS1 survey scan was from 350 to 1500 m/z, and data was acquired at a high resolution of 120,000 (m/z200). The automatic gain control (AGC) target value was 3 × 10^5^ with a maximum ion injection time of 100ms. As for the second stage of mass spectrometry (MS2) scans, charge state 2–7, Dynamic exclusion 50 s, Cycle time 3 s, were selected from the first stage of mass spectrometry (MS1) full scan with an isolation width of 1.6 m/z. The MS2 spectra were acquired on Ion Trap at a rapid scan rate with maximum injection time of 50ms, AGC target 2 × 10^4^ and fragmentation in collision induced dissociation (CID) with normalized collision energy ~ 35%.

### Data analysis

MS raw data files were processed using Proteome Discoverer version 2.2 (Thermo Fisher Scientific, Waltham, MA, USA), and searched against the Homo sapiens UniProt database version 2021 containing 165,800 annotated protein sequences and 51,523,545 residues, which were combined from both sections of the UniProt database, including Swiss-Prot and TrEMBL, using an in-house Mascot server (version 2.5.0; Matrix Science Ltd., London, UK). These sequences were downloaded in July 2021. Additionally, we included a common contaminants database downloaded from the MaxQuant database in December 2020 as part of our search library. Search parameters were as follows: trypsin was chosen as the digestion enzyme, allowing up to two missed cleavage sites per peptide. The MS1 mass tolerance was set to 10ppm, while fragment mass tolerance for MS/MS spectra was set to 0.6Da. oxidation of methionine (M) and deamidation of asparagine (N) and glutamine (Q), were specified as dynamic modifications while carbamidomethylation of cysteine (C) residues was chosen as a static modification. Only Master Proteins (containing at least one unique peptide, and ≥ 2 PSMs), with a 95% confidence interval threshold (p < 0.05, Mascot score ≥ 21) were accepted and included in the analysis.

Statistical analyses, including the determination of differentially regulated proteins and visualization, were performed within Microsoft Excel. Following comparative proteomic profiling and quantitative comparison between in-gel and in-solution groups, the over-representation analyses of gene ontology (GO) terms, including the cellular components, biological process, molecular function, and enriched pathway analysis was performed using FunRich; Functional Enrichment Analysis Tool (www.funrich.org).

## Results

### Protein estimation in perfusate samples

Protein estimation was initially carried out on perfusate samples using a copper-based assay. The protein content of perfusate measured this way was notably high, despite the relatively dilute nature of perfusate. A compatibility issue between perfusate and the copper-based assay was suspected, and a Coomassie-based protein estimation assay was tested as an alternative.

An experiment was conducted using solutions of water and UW-MPS spiked with known quantities of BSA protein, in order to compare expected and measured protein recovery by each method and identify the most accurate protein estimation method for perfusate. Predicted and measured protein concentrations were normalised and the standard scores used to calculate percentage error, indicating the accuracy of each measurement method (Fig. 1B).

While both assays measured the concentration of BSA diluted in water reasonably accurately, with low and similar degrees of error (copper: 17%; Coomassie: 12%), the copper-based assay consistently overestimated the protein concentration of solutions containing UW-MPS. The percentage error between predicted and expected protein concentration was 121.8% and 129% for BSA diluted in perfusate and pure perfusate (with no protein spike) respectively. In comparison, the Coomassie-based assay continued to measure protein concentration accurately, with 6% and 4% error recorded for BSA in perfusate and pure perfusate respectively. From these findings it was established that copper-based assays were inappropriate for measuring perfusate concentration, and the Coomassie-based assay was therefore used for subsequent experiments.

### Identification of proteins in kidney perfusate

A total of 24 human kidney perfusate samples were selected from the COPE COMPARE kidney study cohort and underwent protein estimation by Coomassie-based assay. Concentrations of samples ranged from 0.19 to 1.85 µg/µl (Fig. 1C). From these, 6 samples with enough material were selected and prepared for proteome profiling using both in-gel and in-solution digestion.

MS/MS analysis following gel digestion identified a total of 19,022 peptide-spectrum matches (PSMs), of which 1,369 represented unique peptides. This resulted in the identification of 478 proteins across all 6 samples with > 95% confidence. An average of 332 proteins were identified per perfusate sample.

The same 6 kidney perfusate samples underwent in-solution digestion, which identified a total of 468 proteins, 37,626 PSMs and 2084 unique peptides. An average of 346.2 proteins were identified per sample, ranging from 166 to 397 proteins; 138 (29.4%) were detected in all six samples. A complete overview of all identified proteins is provided by Supplementary Files 1 A and 1B.

### Comparison of in-gel and in-solution digestion of kidney perfusate

To assess the efficiency of the protein digestion in in-solution against the standard gel digestion method, six kidney perfusate samples were selected and 2 aliquots from the same sample underwent both in-gel and in-solution digestion for direct comparison. We found that proteins identified following the latter digestion were associated with a greater number of unique peptides and peptide-spectrum matches (PSMs) per protein on average, compared to those identified following in-gel digestion, and a higher average sequence coverage of identified proteins (Table 1B).

Similar numbers of proteins were identified by both digestion methods but the number of peptides identified by in-solution digestion were almost twice that identified by the gel method (3,109 and 1,721 respectively). The percentage sequence coverage, indicating the proportion of each protein matched to identified peptides sequence by LC-MS/MS, ranged from 1 to 89% for proteins identified following in-gel digestion (average coverage 19.1% per protein), and from 1 to 100% for in-solution digestion (average 27.2% per protein). Of the in-gel-digested proteins, 94% had sequence coverage below 40% and 33% below 10%, whereas for in-solution digested proteins, 78% had < 40% and 22% had < 10% sequence coverage. Only 29 proteins were identified with more than 40% coverage following in-gel digestion, compared to 103 proteins following in-solution digestion. When we looked at proteins identified by one method and not the other, we found no notable difference: 257 proteins were identified by gel digestion and not by in-solution; 247 were identified only by in-solution digestion and not by gel. To check the efficiency of trypsin digestion by both methods, we compared the numbers of proteins that fell within various molecular weight ranges (10-20 kDa, 21-30 kDa, 31-40 kDa etc. up to > 100 kDa). Similar numbers of proteins were found within each mass range for both digestion groups, indicating equally successful digestion by both methods. Average protein and peptide characteristics per sample are presented in Table 1B.

We used Mascot Server (version 2.5.0; Matrix Science Ltd., London, UK) for identification, characterisation and quantitation of proteins in perfusate samples. Mascot scores, indicating the statistical probability of an accurate protein identification from the sequence database, were consistently higher on average for proteins identified following in-solution digestion compared to those identified following in-gel digestion, with the average Mascot score per protein 656.4 for the latter and 1688 for the former (Table 1B). Thus, the results suggest that digestion using in-solution is more reliable compared to the in-gel method.

### Application of the method to other perfusate types

Having demonstrated the efficiency of our in-solution digestion method with kidney perfusion solution, we wanted to show its applicability to other perfusion solutions and perfusion types.

We selected a perfusion solution from the Liver Defatting Study, which represents a significantly different type of perfusate material: this solution was supplemented packed red blood cells and numerous additional pharmacological agents (see methods), and the perfusion process itself took place at normothermic temperature (as opposed to hypothermic in the case of the kidney). The liver itself is also a substantially more metabolically active organ than the kidney, and it was hoped this would be apparent in the resulting protein identifications.

Patient demographics for the two livers included in this study are shown in Table 1 A. We analysed six liver perfusate samples by LC-MS/MS following in-solution digestion and a total of 244 proteins were identified with > 95% confidence from 1,067 distinct peptides derived from 7,208 PSMs (Supplementary Table 1 C). In-gel digestion was then performed on two of the six liver perfusate samples in order to compare the efficacy of the methods against each other in the context of liver perfusion solution. From these, 87 proteins/198 peptides were identified by in-gel digestion, versus 89 proteins/344 peptides identified in the same two samples by in-solution digestion (Supplementary Table 1D). As with the kidney, protein identifications following in-solution digestion were associated with a greater number of peptides, unique peptides and peptide-spectrum matches, suggesting more reliable protein identifications. Sequence coverage of identified proteins ranged from 1 to 55% for in-gel digestion and 2–80% for in-solution. Average sequence coverage was higher for in-solution digestion, at 19.7% per protein, compared to 16% for in-gel digestion (Table 1B). Interestingly, we observed that only 68 proteins were identified by gel digestion and not by in-solution digestion, whereas a much greater 225 were identified following in-solution digestion and not by gel digestion.

### Protein characteristics

**Fig. 2:**
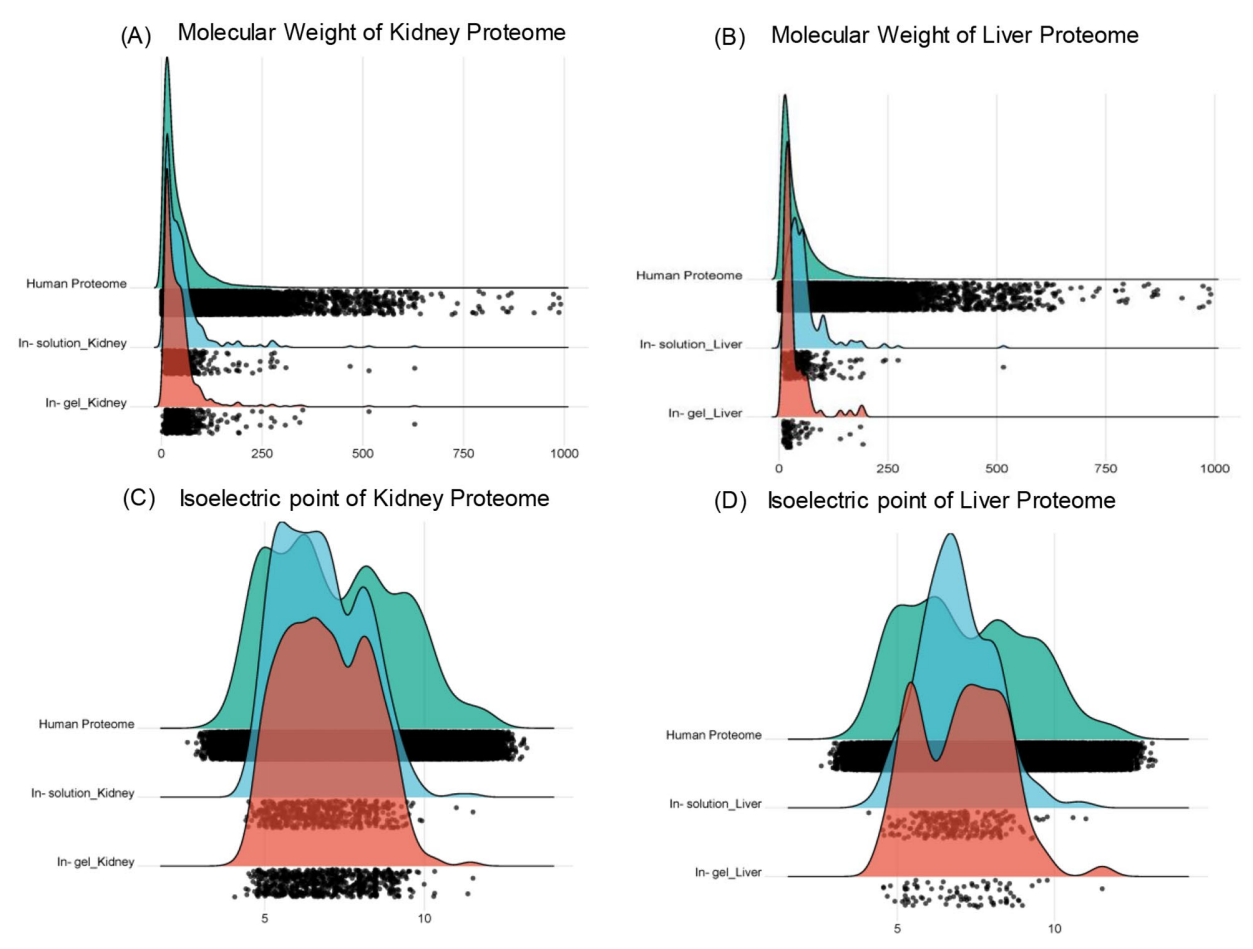
Ridgeline plots showing the distributions of the Molecular weight (kDa) and isoelectric point (pI) of proteins identified in Kidney (**A** and **C**) and liver (**B** and **D**) perfusate samples by in-gel and in-solution digestion methods. Each raincloud dot is the individual protein identified in perfusate samples and proteins present in the human reference proteome

The distribution of proteins based on their molecular weight (MW) and isoelectric point (pI) was evaluated for both sample preparation methods. We observed that the in-solution method allowed us to recover a higher number of larger proteins. The molecular weights of gel-digested proteins ranged from 11.1 kDa to 192.8 kDa, while in-solution digestion identified a broader range of protein weights, from 4.5 kDa to 515.2 kDa, indicating a greater diversity of recovered proteins. Among the proteins identified through gel digestion, 70% fell within the 10–30 kDa range, indicating an overrepresentation of lower molecular weight proteins. In contrast, proteins identified following in-solution digestion were more evenly distributed across the entire weight range (see Fig. 2).

In comparison with the expected distributions based on all proteins present in the human reference proteome (UniProtKB Homo sapiens UP000005640, canonical with 92,158 entries), relatively fewer small and basic proteins were detected by the different methods (see Fig. 2). Mascot scores were once again higher overall in the in-solution digested group compared to gel (see Table 1B).

### Interpretation of proteome changes in kidney and liver perfusate

Having demonstrated the efficiency of the in-solution digestion method in both types of perfusate, we used the data to assess the proteomes of the kidney and liver perfusate in more detail.

Data from 8 kidney perfusate samples that had undergone hypothermic machine perfusion in the presence and absence of oxygen (HMPO_2_ and HMP), for three timepoints: P1 (15 min after the start of perfusion, n = 2), P2 (during perfusion, before leaving the donor centre, n = 2) and P3 (end of perfusion, n = 4) was used. There was an overall increase in the number of proteins identified at timepoints P2 and P3 compared to P1 (Fig. 1D).

**Fig. 3:**
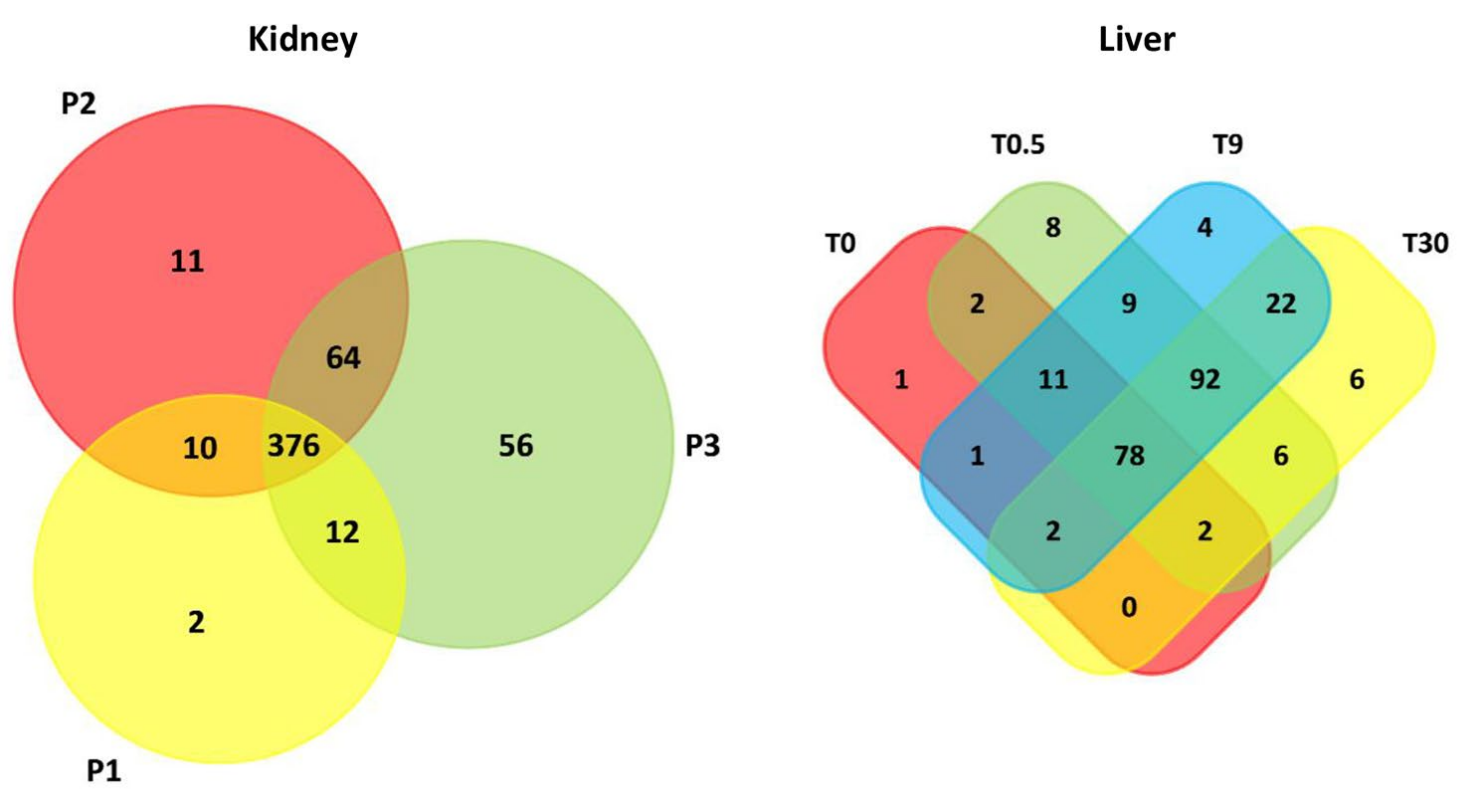
Similarities in proteomic profiles of kidney and liver perfusate samples. Numbers indicate the number of proteins in common between each combination of timepoints

Proteomic profiles were compared between perfusion timepoints. The majority of proteins were identified at all timepoints, with a high degree of similarity also seen between P2 and P3 timepoints. P3 samples had the greatest number of unique proteins not found at any other timepoints (56) (Fig. 3).

In-solution digestion was carried out on six liver perfusate samples in total, taken from two livers (L1 and L2) at 3 timepoints each, from 0 to 30 h perfusion duration (T0-30). It was observed that the average number of proteins identified at each timepoint increased with perfusion duration (Fig. 1E). A large proportion of proteins (78) identified in liver perfuste samples were identified at all timepoints, with an even larger number (92) identified only at timepoints T0.5, T9 and T30, possibly suggesting an uptick in protein secretion triggered by the start of perfusion (Fig. 3).

### Interpretation of biological changes in kidney and liver perfusate

Taking into account the numbers of perfusate proteins identified per sample and the confidence of identification data, we established that the in-solution digestion method shows significant advantages over in-gel digestion. Therefore, only proteins identified by this method were used for Gene Ontology (GO) enrichment analysis. A total of 261 genes from kidney perfusate and 138 genes from liver perfusate were analysed using FunRich analysis software (www.funrich.org).

**Fig. 4:**
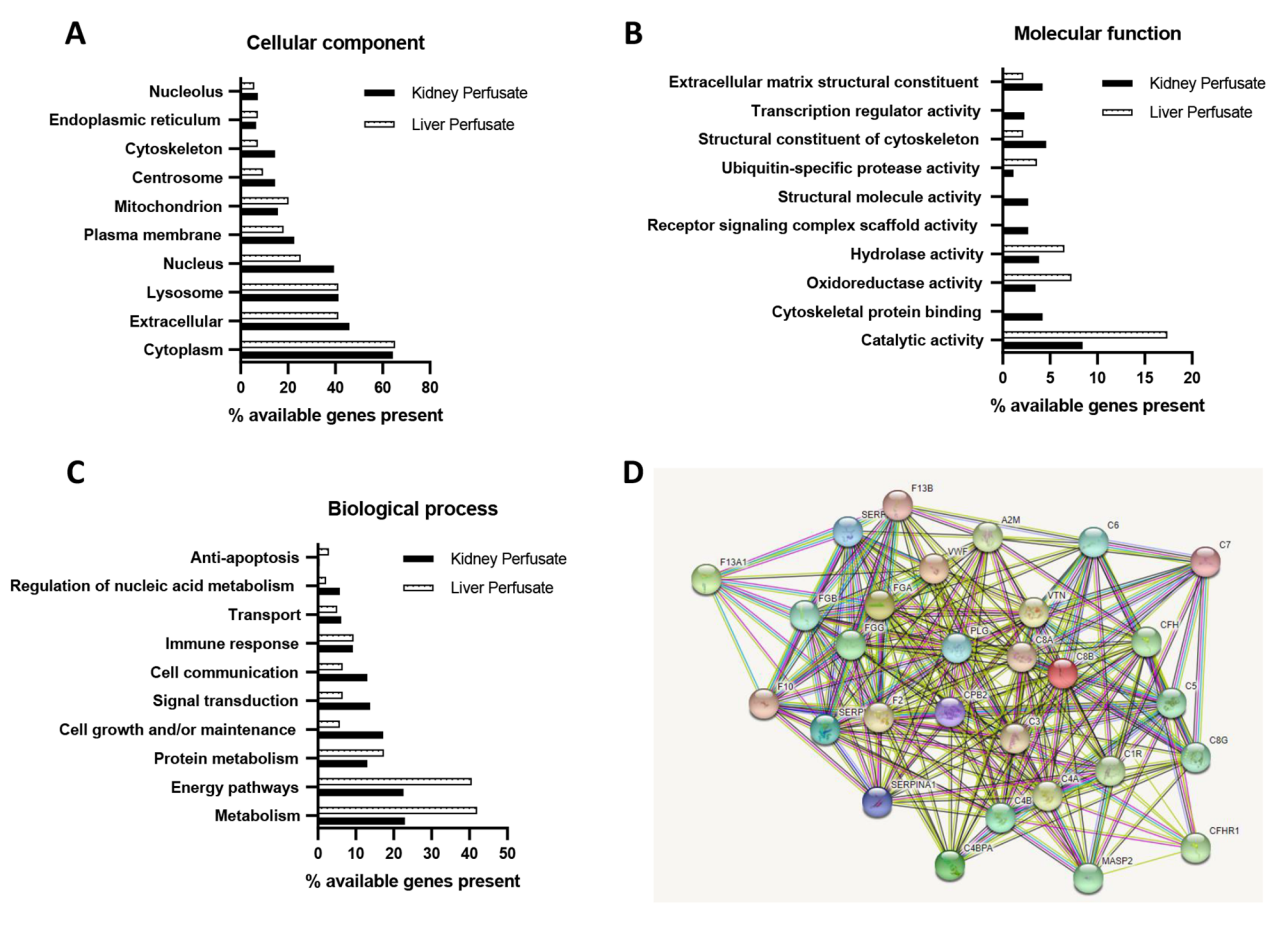
(**A**-**C**) Gene Ontology analysis of perfusate proteins. Number of proteins identified within kidney and liver perfusate datasets expressed as a percentage of total number of available genes in background dataset/database. (**D**) STRING analysis showing interaction network of complement and coagulation cascade proteins in kidney and liver perfusate

Cellular component analysis of kidney and liver perfusate showed similar proportions of cytoplasm, extracellular and lysosome, membrane and Golgi apparatus related genes, (Fig. 4A (Supplementary Table 2 A)). Biological processes enriched within liver perfusate included energy pathways, metabolism, anti-apoptosis and aldehyde metabolism pathways, while kidney perfusate samples were enriched in cell growth and/or maintenance, signal transduction, cell communication, cell growth and regulation of cell cycle (Fig. 4C (Supplementary Table 2B)). The liver perfusate proteome had higher levels of proteins involved in catalytic, oxidoreductase and hydrolase activities, whereas kidney perfusate samples were enriched in proteins involved in cytoskeletal binding, molecular structural activity and transcriptional regulator activity (Fig. 4B (Supplementary Table 2 C)).

A substantial number of the proteins identified in kidney and liver perfusate samples, almost 44% and 51% of their proteomes respectively, have been reported in human plasma (http://www.plasmaproteomedatabase.org/). This included members of the complement and coagulation cascade, of which 14 (A2M, C1R, C3, C4B, C4BPA, C5, C6, F2, FGB, FGG, PLG, SERPIND1, SERPINF2, VTN) were identified in perfusate collected from both kidney and liver perfusions (Fig. 4D), 6 proteins (C4A, C7, C8A, C8B, C8G, CPB2) were unique to kidney perfusate and 2 (CFH and CFHR1) were unique to liver perfusate. Also identified were members of the peroxiredoxin family of antioxidant enzymes (Prx-1, 2, 3, 5 and 6 identified in kidney and liver perfusate) and the high density lipoprotein family (APOA1, APOA2, APOB, APOC3, APOE present in perfusate collected from both organs and APOA1BP, APOA4, APOC4, APOC2 present only in kidney perfusate samples). Two haemoglobin isoforms: HBB and HBD were identified in both perfusate types, while HBA1 was detected in liver and HBA2 in kidney perfusate samples.

A total of 8 genes known to be specifically enriched in the kidney were identified in kidney perfusate samples (https://www.proteinatlas.org/) ATPase H + transporting V1 subunit B1 (ATP6V1B1), crystallin lambda 1 (CRYL1), dimethylarginine dimethylaminohydrolase 1 (DDAH1), fructose-bisphosphatase 1 (FBP1), glutathione peroxidase 3 (GPX3), lactate dehydrogenase B (LDHB), phosphotriesterase-related (PTER), and uromodulin (UMOD) while a total of 93 gene products identified in liver perfusate samples have high expression in liver compared to other organs. Among those, 19 genes are already FDA approved drug targets, such as coagulation factor II, thrombin (F2), acetyl-CoA acyltransferase 1 (ACAA1), alcohol dehydrogenase 1 A (ADH1A), aldehyde dehydrogenase 2 (ALDH2), aminolevulinate dehydratase (ALAD), catalase (CAT), fibrinogen beta chain (FGB), fibrinogen gamma chain (FGG), plasminogen (PLG), and guanidinoacetate N-methyltransferase (GAMT).

## Discussion

Proteomic profiling of perfusion fluid represents an exciting opportunity to understand some of the biological, cellular and metabolic processes underlying organ preservation and transplantation, however, to date little has been reported on the best methods of preparing perfusate for analysis by mass spectrometry. In this study, we compared methods of protein estimation and digestion in order to identify the most effective in relation to kidney and liver perfusate from hypothermic and normothermic organ perfusions. From this, it was established that in-solution digestion is better than in-gel digestion, based on the numbers of proteins and peptides identified, along with other parameters to measure reliability and confidence in individual peptide and protein IDs. We went on to look more closely at some of the specific protein families identified, and linked them to pathways that play key roles in the transplantation process.

When preparing samples for proteomic analysis, choosing an accurate and compatible protein estimation assay is an important first step. This is especially the case in situations such as organ transplant, where the biological material being analysed has immense significance on the viability for life saving treatment. By comparing the copper-based and Coomassie-based assays it was established that the copper-based assay is inaccurate in measuring protein concentration in the presence of UW-MPS perfusate. The reason for this is likely the presence of reduced glutathione, an antioxidant tripeptide, which is included in UW-MPS for its ROS-scavenging properties [20]. Glutathione is made of three amino acids, including cysteine, one of the peptides responsible for the reducing reaction that drives the colour change in the copper-based assay [21]. Its presence in perfusate is therefore likely to elicit a strong colour change in the sample solution, regardless of protein content, resulting in an increased and inaccurate apparent protein concentration [22, 23]. In contrast, the Coomassie-based assay relies upon Coomassie Brilliant Blue dye, which undergoes a shift in absorbance when bound to proteins [24]. This shift is not impacted by specific peptide residues, reducing or chelating agents and indeed the assay is compatible with most substances [24], therefore, when used to measure perfusate concentration, the results were more accurate.

The next step of the sample preparation process, protein digestion and LC-MS/MS analysis of peptides recovered from a set of SDS-PAGE gel bands, has long been a common method used in LC-MS/MS workflows [25]. The in-gel digestion approach is useful for allowing complex samples to be split up prior to analysis; dividing a single sample up into sections will reduce its complexity and allow each run to perform a deeper analysis of the protein mixture, potentially leading to more protein identifications and helping mitigate the effects of the large dynamic range of proteins found in perfusate or other samples [17]. It also provides an additional purification step for samples that may still contain contaminants such as buffers or detergents, as well as the opportunity to remove the highly abundant blood albumin protein from samples, in the hope of limiting its masking effects and improving the detection of low abundance proteins [26]. So far there is only one published example of unbiased gel digestion of human kidney perfusate: Van Leeuwen et al. identified a total of 300 proteins with at least one unique peptide in 44 samples [18], while our in-gel analysis method identified 478 proteins in 6 kidney perfusate samples irrespective to sample collection timepoints.

For both kidney and liver samples, in-solution digestion produced protein identifications that were associated with higher MASCOT scores and greater numbers of PSMs, unique peptides and percentage coverage, all of which indicate a more reliable identification. The larger number of unique peptides and higher sequence coverage, on average, compared to in-gel digestion, indicated that the in-solution digestion method provides more opportunities for unique proteins to be identified that may only be present in a few samples. Additionally, in-solution digestion identified an overall greater number of proteins. Similar findings were reported by Klont et al. when comparing in-solution digestion with in-gel, on-filter, and on-pellet digestion methods for three different otolaryngeal tissue types: nasal polyps, parotid gland, and palatine tonsil [27]. The group reported an improved yield of peptides extracted via this method, making it a viable option for quantitative proteomics with limited losses and good precision for peptide and protein quantification. They also reported the recovery of additional peptides and found no bias regarding the amino acid composition or physicochemical properties of the identified peptides and proteins when compared to other methods [27]. These findings support our conclusions that in-solution digestion is useful for identifying a larger number of proteins with greater reliability. The same study also reported higher abundances of high molecular weight proteins in the solution-digested group [27], a finding supported by our data, which identified a wider range of molecular weights following in-solution digestion, especially at the higher MW end. A different study that aimed to compare in-solution and in-gel digestion methods from a quantitation perspective reported both low and highly variable peptide yields from gel digests compared to solution digests, with up to a 50% error reported between the recovered peptide yield and the amount of protein used in the analysis. Meanwhile, they reported that in-solution digestion produced peptide yields comparable to what was expected [28].

We have not directly quantified or compared peptide yield between methods in this experiments, but this could be an interesting future investigation. Sample quantity and availability can frequently be a limiting factor when using material obtained from clinical trials, so a method that maximises peptide yield and minimises sample wastage will be particularly beneficial.

The proteins that were identified only by the in-solution method do not show any obvious physico-chemical differences compared to proteins identified by in-gel digestion. These proteins were likely not identified by the latter method due to a combination of less efficient peptide extraction and random ‘picking’ of peptide peaks for sequencing by data analysis software.

Despite the popularity of gel digestion, it remains a laborious and error-prone process and carries risks of reduced peptide recovery compared to in-solution digestion [25, 29]. The process is lengthy and multi-step, allowing ample opportunity for peptides to be lost and contamination to be introduced, especially when sectioning of the gel is carried out by hand rather than with a spot-picker. Speicher et al. reported adsorptive loss of peptides resulting from the use of plasticware such as pipettes and microcentrifuge tubes [30]. This is likely to happen to a greater degree during gel digestion due to the high number of washes and increased handling of liquid peptide extracts with pipette tips. They also observed 25–50% peptide loss when acetonitrile was used for peptide extraction and the Speedvac used to dry samples, and found that as well as reducing peptide recovery, these actions led to a high degree of variability in recovery within and between experiments, even when identical methods were used [30]. There is also a risk of low molecular weight proteins, especially those present in small quantities, running too far down the gel or not taking up enough stain to produce a visible spot to be excised, meaning these proteins may be unintentionally discarded and not identified during the analysis. Similarly, the strongly stained albumin band was intentionally excluded from gel digestion to avoid masking effects, but the nature of doing this by hand means that additional proteins of similar molecular weights may have been removed at the same time. It has also been suggested that large peptides in particular may become trapped in the gel matrix and/or may be inaccessible by proteases such as trypsin, meaning they do not get digested as efficiently as when they are in solution [31]. Finally, while gel LC-MS/MS may be convenient for metabolic labeling strategies like TMT or ITRAQ, it presents gel-slice reproducibility issues for chemical labeling and label-free approaches, whereas In-solution digestion can be consistently and widely applied.

We undertook a panoramic profiling approach to the proteome analysis carried out in this study, with the aim of identifying and assessing the widest range of proteins possible, including those expressed at lower levels or only in a subset of patient samples. Given the complexity of the cellular and physiological events underlying the transplant process, and the natural heterogeneity that exists in sample populations and their proteomes, it is necessary to study a broad spectrum of proteins. We also hope that a more exploratory, rather than targeted, approach to proteome investigation may lead to the identification of biomarker candidates not previously considered in the context of transplantation, which could ultimately lead to the identification of new therapeutic targets.

Upon more detailed investigation of the perfusate proteome, we observed the presence of a number of plasma-derived proteins. The intention of analysing perfusate is to identify proteins secreted by the organ during the perfusion process, so this was a promising and interesting finding. A number of the identified protein families were of interest due to the roles they play in the physiological response to organ transplantation and, in particular, the development of ischaemia-reperfusion injury.

Multiple members of the complement and coagulation cascade were identified in our samples. The complement system forms part of the innate immune response and consists of a variety of plasma-derived and cell surface proteins which are naturally found throughout the body. Exposure to pathogens activates these proteins and, via an enzyme-triggered cascade, results in the generation of a large number of effector molecules that play roles in inflammation, phagocytosis and destruction of bacterial walls [32]. Studies show that ischaemia-reperfusion injury (IRI) can activate the complement cascade, and complement inhibitors have shown protective effects from IRI in pre-clinical models, so it remains an important area of study in the context of allograft injury [33]. Also identified in both kidney and liver perfusate samples were members of the high density lipoprotein (HDL) family. HDLs, often described as “good cholesterol”, have systemic anti-oxidant and anti-inflammatory properties which can protect against tissue damage and protomote endothelial repair and regeneration [34]. They are well known for the benefits they provide in reducing the risk of cardiovascular disease and have been shown to reduce the risk of acute kidney injury following cardiac surgery. They can also protect against IRI in the kidney and liver [34]. Peroxiredoxins (Prxs) are a ubiquitous family of antioxidant enzymes which regulate peroxide levels in order to protect against oxidative stress, support metabolism and contribute to cell signalling pathways [35, 36]. Of these, low levels of Prx3 have been associated with chronic kidney injury in mice [37], while levels of Prx1, 2, 3 and 4 have been described as biomarkers of oxidative stress in renal disorders that precede chronic kidney disease [38]. Prxs also mediate the liver’s response to acute and oxidative damage and are used as biomarkers of inflammatory liver disease and IRI [39].

Of the kidney-enriched proteins, glutathione peroxidases play a detoxification role in the body, protecting cells from oxidative damage by destroying hydrogen peroxide [40]. GPx3 is present in the extracellular fluid and plasma, meaning it can be easily accessed and assayed, making it a valuable biomarker candidate [40]. High levels of UMOD have previously been linked to increased estimated glomerular filtration rate (eGFR), and more recently to potentially reduced risk of acute kidney injury [41].

Of the liver-enriched proteins, coagulation factor II (also known as prothrombin) is synthesized in a pre/pro form by liver hepatocytes and undergoes numerous post-translational modifications before the active mature product, thrombin, is released into the plasma [42]. Thrombin supports the process of blood clotting by converting fibrinogen to fibrin, activating platelets and driving vascular remodelling following endothelial injury. Due to its important role in these processes, it is a popular target for anti-coagulation therapies [42]. Catalase (CAT) is another enzyme involved in protection from oxidative damage by free radicals, which it does by converting hydrogen peroxide into water [43]. Levels of CAT, and other antioxidant enzymes such as SOD (superoxide dismutase) and GSH (reduced glutathione, mentioned above) were reduced in patients with liver cirrhosis or undergoing liver transplantation for other reasons [44].

Finally, we identified potential biomarkers in the context of ischemia reperfusion injury and delayed graft function in the kidney perfusate proteome. These included members of the annexin family (ANXA1, ANXA2, ANXA3 and ANXA5), of which ANXA2 has been reported as a potential candidate [45]. We also identified a number of proteins being investigated as post-transplant indicators for acute rejection: pigment epithelium-derived factor 1 (SERPINF1) and ncotinamide phosphoribosyltransferase (NAMPT) in both perfusate types, brain acid soluble protein 1 (BASP1) only in kidney and nicotinate phosphoribosyltransferase (NAPRT) only in liver perfusate samples [45].

Our experiments demonstrated that despite the historic popularity and continuing development of the in-gel digestion methodology [46–49], in-solution digestion is equally, if not more, successful at detecting the proteins in a perfusion sample. In-gel digestion is laborious, time-consuming and has more chance of error and sample loss whereas in-solution digestion provides the opportunity for unbiased and thorough peptide extraction, digestion and detection with minimal loss. To avoid any mis-cleavage bias of trypsin in urea buffer, a sequential Lys-C/trypsin digestion step can be used [50]. In-solution digestion is overall much simpler than gel digestion, and less hands-on time is required, therefore more samples can be digested at a time in this way.

## Conclusion

Perfusate obtained throughout the transplant preservation processes hold a potential wealth of proteomic information that may aid in determining organ quality, optimising treatment protocols and predicting transplant outcomes. Up to now, there has not been a definitive set of guidelines for the preparation of perfusate for mass spectrometry analysis. We have confirmed the in-solution digestion method to be a straightforward, efficient and unbiased digestion protocol on both the protein and peptide level for use in proteome profling of perfusate in solid organ transplantation. As novel and updated methods are likely to emerge, our workflow may serve as benchmark for future studies aiming to objectively run proteomic analysis of perfusate.

### Electronic supplementary material

Below is the link to the electronic supplementary material.

Supplementary Material 1

Supplementary Material 2

Supplementary Material 3

## Data Availability

The mass spectrometry proteomics data have been deposited to the ProteomeXchange Consortium via the PRIDE [https://www.ebi.ac.uk/pride/archive] partner repository with the dataset identifier PXD040856.
