# Supplementary material for: Comparison of in-gel and in-solution proteolysis in the proteome profiling of organ perfusion solutions
Source: Clin Proteomics. 2023 Nov 15;20:51. doi: 10.1186/s12014-023-09440-x (PMC10648346; doi:10.1186/s12014-023-09440-x)
Supplement: Supplementary file 2 — Supplementary Material 2 [file 12014_2023_9440_MOESM2_ESM.docx]

**Supplementary figure 1: SDS-PAGE analysis of kidney and liver perfusate samples**

**A**: Guidelines for dividing SDS-PAGE lanes into sections according to Mw prior to in-gel digestion; **B-D**: Kidney perfusate samples SDS-PAGE (20ug/lane); **E-F**: Liver perfusate samples SDS-PAGE (20ug/lane)

M: marker; KP: kidney perfusate; C: cell line control (PBMC); PB: perfusate blank; LP: liver perfusate; T: timepoint (hours); -/+: before/after ethanol depletion; BB: buffer blank

**Supplementary figure 2: similarity of perfusate proteomes**

Heatmaps comparing proteomes of individual In-solution digested perfusate samples against each other. **A**: kidney perfusate; **B**: liver perfusate.


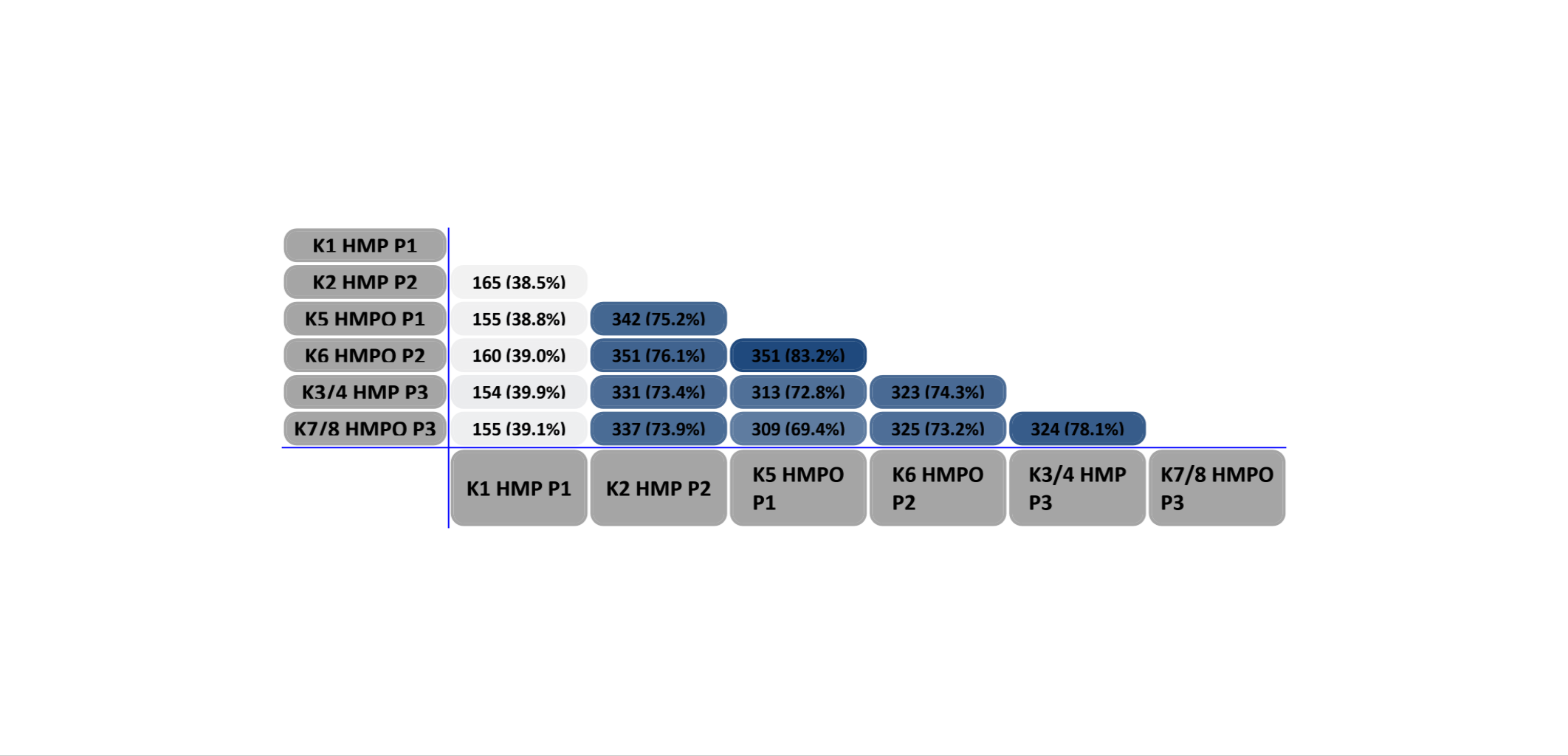

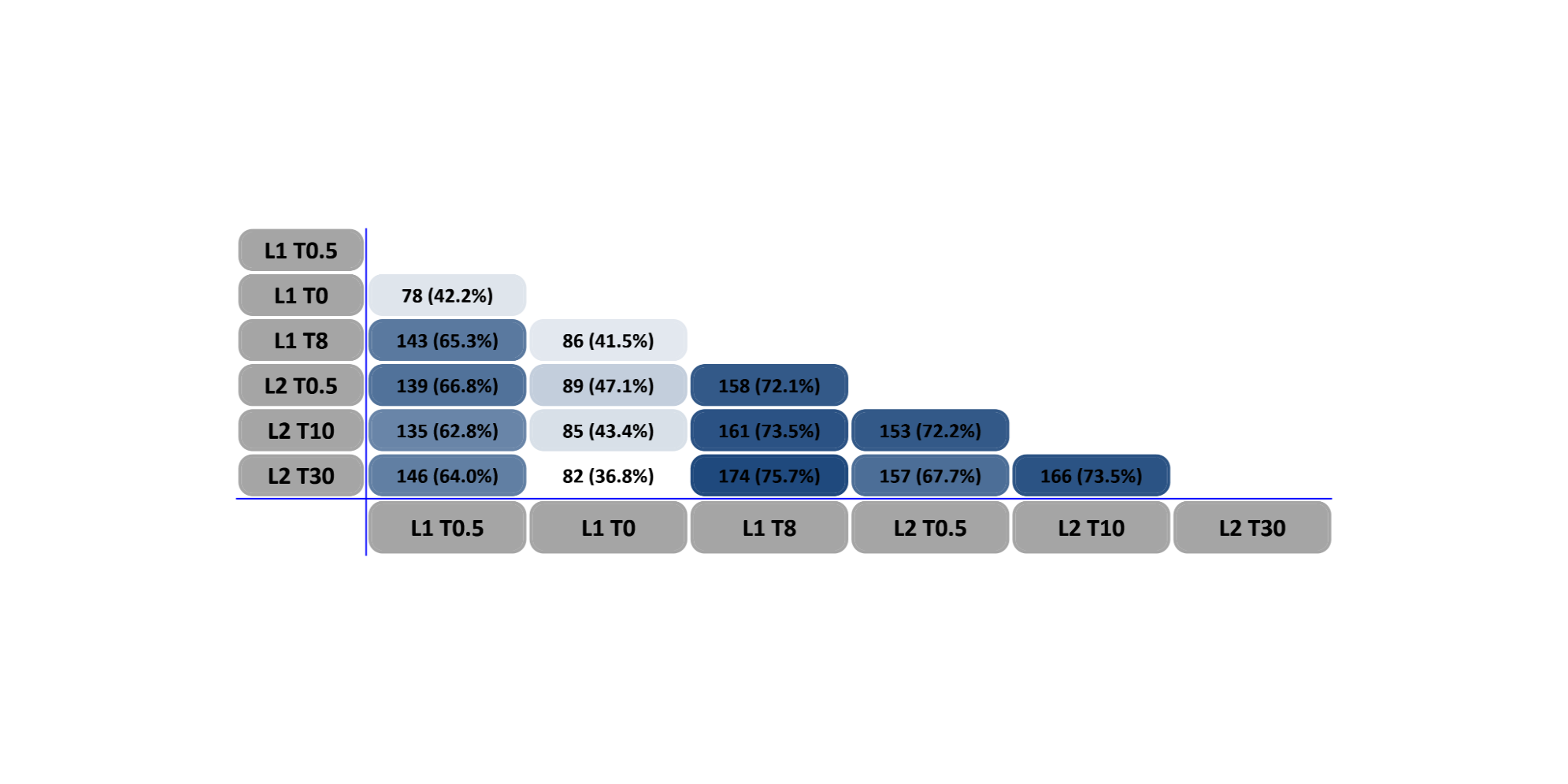


**A**

**B**
